# Heterologous Production of a Novel Cyclic Peptide Compound, KK-1, in *Aspergillus oryzae*

**DOI:** 10.3389/fmicb.2018.00690

**Published:** 2018-04-09

**Authors:** Akira Yoshimi, Sigenari Yamaguchi, Tomonori Fujioka, Kiyoshi Kawai, Katsuya Gomi, Masayuki Machida, Keietsu Abe

**Affiliations:** ^1^ABE-Project, New Industry Creation Hatchery Center, Tohoku University, Sendai, Japan; ^2^Kumiai Chemical Industry Co., Ltd., Tokyo, Japan; ^3^Laboratory of Bioindustrial Genomics, Department of Bioindustrial Informatics and Genomics, Graduate School of Agricultural Science, Tohoku University, Sendai, Japan; ^4^Bioproduction Research Institute, National Institute of Advanced Industrial Science and Technology (AIST), Tsukuba, Japan; ^5^Laboratory of Applied Microbiology, Department of Microbial Biotechnology, Graduate School of Agricultural Science, Tohoku University, Sendai, Japan; ^6^Department of Microbial Resources, Graduate School of Agricultural Science, Tohoku University, Sendai, Japan

**Keywords:** antifungal activity, *Aspergillus oryzae*, gene cluster, heterologous production, non-ribosomal peptide

## Abstract

A novel cyclic peptide compound, KK-1, was originally isolated from the plant-pathogenic fungus *Curvularia clavata*. It consists of 10 amino acid residues, including five *N*-methylated amino acid residues, and has potent antifungal activity. Recently, the genome-sequencing analysis of *C. clavata* was completed, and the biosynthetic genes involved in KK-1 production were predicted by using a novel gene cluster mining tool, MIDDAS-M. These genes form an approximately 75-kb cluster, which includes nine open reading frames, containing a non-ribosomal peptide synthetase (NRPS) gene. To determine whether the predicted genes were responsible for the biosynthesis of KK-1, we performed heterologous production of KK-1 in *Aspergillus oryzae* by introduction of the cluster genes into the genome of *A. oryzae*. The NRPS gene was split in two fragments and then reconstructed in the *A. oryzae* genome, because the gene was quite large (approximately 40 kb). The remaining seven genes in the cluster, excluding the regulatory gene *kkR*, were simultaneously introduced into the strain of *A. oryzae* in which NRPS had already been incorporated. To evaluate the heterologous production of KK-1 in *A. oryzae*, gene expression was analyzed by RT-PCR and KK-1 productivity was quantified by HPLC. KK-1 was produced in variable quantities by a number of transformed strains, along with expression of the cluster genes. The amount of KK-1 produced by the strain with the greatest expression of all genes was lower than that produced by the original producer, *C. clavata*. Therefore, expression of the cluster genes is necessary and sufficient for the heterologous production of KK-1 in *A. oryzae*, although there may be unknown factors limiting productivity in this species.

## Introduction

Fungal secondary metabolites and their derivatives often show potent biological activity and have been exploited in various applications, including as medical and agrochemical precursors and intermediates. The secondary metabolites in fungi are usually synthesized by enzymes encoded by clusters of coordinately regulated genes (i.e., in many cases a transcription factor can be associated with a gene cluster, and transcription of the clustered genes is co-regulated by the transcription factor). In addition, most of these clusters encode enzymes such as polyketide synthase (PKS) and non-ribosomal peptide synthetase (NRPS), which catalyze the condensation reactions of monomeric units to form oligomeric intermediates (Keller et al., 2005). The recent decoding of the whole genome sequences of filamentous fungi has greatly contributed to facilitating the identification of numerous genes responsible for the production of such secondary metabolites. For example, the gene cluster involved in kojic acid biosynthesis in the genome of the koji mold *Aspergillus oryzae* was identified on the basis of genome-wide profiling using transcriptome information (Terabayashi et al., 2010). In addition, the gene cluster involved in the biosynthesis of FR901469, which is an inhibitor of β-1,3-glucan synthase and exerts antifungal activity against *A. fumigatus* and *Candida albicans* both *in vitro* and *in vivo* (Fujie et al., 2000a,b), was identified on the basis of molecular structure and motif prediction for the NRPS gene (Matsui et al., 2017). Nowadays, bioinformatics tools, such as SMURF (Khaldi et al., 2010), antiSMASH (Medema et al., 2011; Blin et al., 2013), and CLUSEAN (Weber et al., 2009), have been developed to identify potential secondary metabolites in filamentous fungi. Moreover, another novel program, which is independent of the known sequence motifs of core genes for potential secondary metabolites and is named MIDDAS-M (motif-independent *de novo* detection algorithm for secondary metabolite biosynthetic gene clusters), has been constructed to predict the gene clusters required for secondary metabolite production (Umemura et al., 2013). Using this program, Umemura et al. (2014) discovered a gene cluster that was involved in the biosynthesis of ustiloxin B in the genome of *A. flavus*, which has neither PKS nor NRPS. With the development of such a powerful bioinformatics tool, it is expected that many effective precursors of medical and agricultural chemicals will be discovered in filamentous fungi.

Fungal secondary metabolites have also been studied in relation to the toxins involved in plant pathogenesis (Desjardins and Hohn, 1997). For example, three kinds of phytotoxins produced by *Cochliobolus* species—a cyclic tetrapeptide HC-toxin from *C. carbonum*, a linear polyketide T-toxin from *C. heterostrophus*, and a chlorinated cyclic pentapeptide victorin from *C. victoriae—*are the primary causal agents of northern leaf blight of maize, southern leaf blight of maize, and Victoria blight of oats, respectively (Desjardins and Hohn, 1997). Other examples of toxic fungal secondary metabolites are the ergot alkaloids (Gerhards et al., 2014). The fungus *Claviceps purpurea* is well known as a producer of ergot alkaloids. This fungus is able to infect rye and other grains and has caused several disease outbreaks due to the feeding of rye products contaminated with *C. purpurea* sclerotia (Haarmann et al., 2009; Schiff, 2006). This disease is called ergotism or St. Anthony’s fire (Schiff, 2006; Haarmann et al., 2009; Jakubczyk et al., 2014). A gene cluster involved in the biosynthesis of ergot alkaloids has been identified in *C. purpurea*; the cluster contains 14 genes, including several NRPS genes (Haarmann et al., 2009; Gerhards et al., 2014; Jakubczyk et al., 2014). Because the ergot alkaloids show strong bioactivity, they can act as potent drugs. Methylergometrine is used as a hemostatic agent after childbirth, and ergotamine is used as a migraine medication (Schiff, 2006; Haarmann et al., 2009; Gerhards et al., 2014; Jakubczyk et al., 2014). These are examples of the effective use of fungal secondary metabolites in pharmaceutical applications.

**FIGURE 1:**
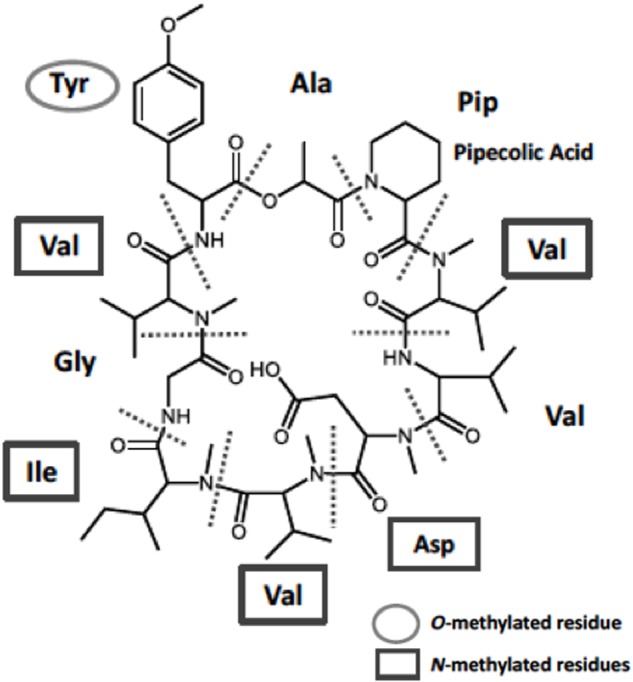
Chemical structure of KK-1 identified in the fungus *Curvularia clavata*. Constituent amino-acid units are separated by the dotted lines: Pip, pipecolic acid residue (non-proteinogenic amino acid); Val, valine residue; Asp, aspartic acid residue; Ile, isoleucine residue; Gly, glycine residue; Tyr, tyrosine residue.

CAS No. 143380-71-6, namely KK-1 (**Figure 1**), is a novel cyclic peptide compound with potent antifungal activity. It was originally identified in the plant-pathogenic fungus *Curvularia clavata* (Sigenari Yamaguchi et al., Kumiai Chemical Industry Co., Ltd., Unpublished results). The teleomorphic state of the *Curvularia* genus is classified mostly into the genus *Cochliobolus* (Manamgoda et al., 2011). Although to our knowledge there has been no report of the phytotoxicity of KK-1, this compound may be involved in the survival strategy of this fungus. KK-1 consists of 10 amino acid residues, including five *N*-methylated residues and one *O*-methylated residue (**Figure 1**). It has potent activity against many plant pathogenic fungi, including the economically important pathogen *Botrytis cinerea* (Yamaguchi et al., Unpublished results). Recently, genome sequence analysis was completed in *C. clavata* (Yamaguchi et al., Unpublished results), and the KK-1 biosynthetic gene cluster was identified in *C. clavata* by using the MIDDAS-M method to predict gene clusters from transcription data (Umemura et al., 2013; Yamaguchi et al., Unpublished results). The cluster contains nine genes (DDBJ accession no. LC371755), including *kkR* (which encodes a basic leucine zipper transcription factor), *OMT* (a gene encoding *O*-methyltransferase), and a large gene (39 kb) encoding NRPS. However, it is not known whether these cluster genes are necessary, or sufficient, for the biosynthesis of KK-1 in *C. clavata.* Thus, heterologous production by introducing the cluster genes into other fungal hosts might be an effective strategy for identification of the genes necessary for the biosynthesis of KK-1.

The koji mold *A. oryzae* is used to produce traditional Japanese fermented foods such as *sake* (rice wine), *shoyu* (soy sauce), and *miso* (soybean paste) (Machida et al., 2008). In addition, *A. oryzae* is important for producing a variety of substances in the fermentation industry worldwide. These products have been widely applied in numerous industries, including the food, chemical, and pharmaceutical industries (Abe et al., 2006). Although *A. oryzae* is closely related to *A. flavus*, which produces many secondary metabolites, including aflatoxin and aflatrem (the most potent natural carcinogen and a tremorgenic mycotoxin, respectively), *A. oryzae* does not produce any toxic metabolites because its secondary metabolite genes are silenced (Machida et al., 2005). For instance, homologs of the aflatoxin biosynthesis gene cluster are not expressed in *A. oryzae*, even under conditions favorable for aflatoxin production in *A. flavus* and *A. parasiticus* (Kusumoto et al., 1998; Watson et al., 1999; Takahashi et al., 2002; Zhang et al., 2005). Therefore, *A. oryzae* may be a preferable host for the production of not only heterologous proteins but also secondary metabolites with important medical properties (Kobayashi et al., 2007; Machida et al., 2008; Sakai et al., 2008).

Here, we performed heterologous production of KK-1 in *A. oryzae*: we introduced cluster genes into the genome of *A. oryzae* to determine whether the cluster genes predicted in *C. clavata* were responsible for the biosynthesis of KK-1. Because the NRPS gene was large (approximately 40 kb) and it was not known whether such a large heterologous gene could be introduced into, and function in, the *A. oryzae* genome, the gene was split into two fragments and then reconstructed in the *A. oryzae* genome. The remaining seven genes (excluding the *kkR* gene) were simultaneously introduced into the *A. oryzae* strain to which NRPS had been introduced. To evaluate the heterologous production of KK-1 in *A. oryzae*, gene expression was analyzed by means of the reverse transcription–polymerase chain reaction (RT-PCR) and KK-1 productivity was analyzed by means of high-performance liquid chromatography (HPLC). We succeeded in producing KK-1 in *A. oryzae*, along with heterologous expression of the cluster genes. To the best of our knowledge, the KK-1 NRPS gene is the largest heterologously expressed gene in *A. oryzae*. We also compare and discuss the levels of production of KK-1 in *C. clavata* and *A. oryzae*.

## Materials and Methods

### Strains and Growth Media

An auxotrophic *adeA* mutant of *A. oryzae* (Δ*ligD*::*sC*, *niaD*^-^, Δ*adeA::ptrA*) (Zhang et al., 2017) was used as the recipient strain for heterologous introduction of the KK-1 biosynthetic gene cluster. The *A. oryzae* CNT (Δ*ligD*::*sC*, *niaD*^-^, Δ*adeA::ptrA*, *adeA*^+^) strain was used as a control for gene expression and KK-1 production. All *A. oryzae* strains were cultured in CDE medium, which is Czapek Dox (CD) medium (Nakajima et al., 2000) in which the nitrogen source is 70 mM sodium hydrogen L(+)-glutamate monohydrate instead of 70 mM NaNO_3_ (Mizutani et al., 2008). CDEAX medium, which contained 0.01% (w/v) adenine and 1% xylose instead of 2% glucose in CDE medium, was used for marker recycling in *A. oryzae*. Conidia of *A. oryzae* were isolated from cultures grown on malt medium containing 9% (w/v) malt extract (Becton Dickinson and Company, Sparks, MD, United States), 0.5% (w/v) yeast extract (Becton Dickinson and Company), and 0.1% (w/v) trace element solution (Miyazawa et al., 2016). YPM medium (1% yeast extract, 2% polypeptone, 2% maltose) was used to upregulate the *amyB-*promoter-driven genes introduced into the *A. oryzae* strain.

### Construction of Vectors for KK-1 Reconstruction in *A. oryzae*

Vector plasmids for KK-1 reconstruction in *A. oryzae* were constructed as follows (Supplementary Figure S1). The front half of the NRPS gene of *C. clavata* (approximately 20 kb) was amplified by using PrimeSTAR GXL DNA polymerase (TAKARA, Tokyo, Japan) and the primers NRPS_FH-F-Not I and NRPS_FH-R-Not I (Supplementary Table S1). Each primer was designed to introduce a *Not*I site. *C. clavata* genomic DNA was used as the template. The amplified fragment was cloned into the *Eco*RV (blunt end) site of pZErO-2 (Invitrogen-Thermo Fisher Scientific, Waltham, MA, United States) (Supplementary Figure S1). Then, the fragment containing the front half of the NRPS gene was digested with *Not*I, corrected, and inserted into the *Not*I site of pAAG-Cre (Supplementary Figure S1), in which both the selectable marker for *A. oryzae* (the *A. nidulans adeA* gene) and the Cre recombinase gene (*cre*) conditionally expressed by the xylanase-encoding gene promoter were designed to be located between the mutant *lox* sequences *lox66* and *lox71* (Zhang et al., 2017). The plasmid was constructed by replacing the *amyA* terminator of pAAAXG-Cre with the *glaA* terminator (Zhang et al., 2017). The sequences of the resulting plasmid, pAAGC-NRPSfh (Supplementary Figure S1), were confirmed, and the plasmid was used to transform the *A. oryzae* Δ*adeA* strain (*sC*^-^, *niaD*^-^, Δ*ligD*::*sC*, Δ*adeA*::*ptrA*), as described previously (Fujioka et al., 2007).

Because (for unknown reasons) the rear-half fragment of the NRPS gene of *C. clavata* could not be cloned directly into the plasmid pAAG-Cre, by using the same strategy as for NRPSfh, In-Fusion Cloning technology (Clontech, Mountain View, CA, United States) was used (Supplementary Figure S2). Three fragments, designated NRPS rh fragment A, NRPS rh fragment B, and NRPS rh fragment C, were amplified by using PrimeSTAR GXL DNA polymerase with the primers NRPS_RH-IF1-Not I-F and NRPS_RH-IF1-R (for fragment A), NRPS_RH-IF2-F and NRPS_RH-IF2-R (for fragment B), and NRPS_RH-IF3-F and NRPS_RH-IF3-Not I-R (for fragment C). The amplified fragments were purified by gel extraction using an illustra GFX PCR DNA and Gel Band Purification Kit (GE Healthcare UK Ltd., Little Chalfont, Buckinghamshire, England) and fused with the *Not*I-digested pAAG-Cre by using an In-Fusion HD Cloning Kit (Clontech) in accordance with the manufacturer’s instructions. The sequences of the resulting plasmid (pAAGC-NRPSrh) were confirmed, and the plasmid was used to transform the NRPSfh-integrated strain of *A. oryzae*.

### Construction of Vectors for Seven Cluster Genes

To introduce the remaining seven cluster genes (i.e., all cluster genes except the gene for NRPS and the gene encoding the transcription factor), we constructed three vector plasmids: pATR0203, pATR678, and pATR09OMT. These plasmids were constructed as follows. The expression of all genes was driven by the *amyB* promoter of *A. oryzae* and terminated by the *amyB* terminator of *A. oryzae*. The plasmid pA3AXPC (Katsuya Gomi et al., Tohoku University, Unpublished), which was constructed on the basis of the plasmid pAAAXP-Cre (Zhang et al., 2017) and harbored a triple-gene expression cassette for overexpression of multiple foreign genes, was used as the gene expression vector for *A. oryzae*. Because the plasmid was capable of carrying three genes, all of which could be regulated by the *amyB* promoter, three vectors, each containing two or three cluster genes, were constructed. The fragments of the seven cluster genes of *C. clavata* were amplified by using PrimeSTAR DNA polymerase (TAKARA) from the cDNA of *C. clavata* as a template. The open reading frame (ORF) fragment of the *TR02* gene was amplified by using the primers TR02-SpeI-F and TR02-SpeI-R (Supplementary Table S1). The ORF fragment of the *TR03* gene was amplified by using the primers TR03-NotI-F and TR03-NotI-R (Supplementary Table S1). The amplified fragments were cloned once into the *Eco*RV (blunt end) site of pZErO-2, and the plasmids were designated pZTR02 and pZTR03, respectively. Then, pZTR02 and pZTR03 were digested with *Spe*I and *Not*I, respectively, to isolate the ORFs of the TRAF02 and TRAF03 genes, respectively. The gene fragments were sequentially inserted into the *Spe*I (*TR02*) and *Not*I (*TR03*) sites of pA3AXPC. The sequences of the resulting plasmid were confirmed and the plasmid was designated pA0302.

The ORF fragment of the *TR06* gene was amplified by using the primers TR06-NheI-F and TR06-NheI-R, and the ORF fragment of the *TR07* gene was amplified by using the primers TR07-NotI-F and TR07-NotI-R (Supplementary Table S1). The ORF fragment of the *TR08* gene was amplified by using the primers TR08-SpeI-F and TR08-SpeI-R (Supplementary Table S1). The amplified fragments were cloned once into the *Eco*RV (blunt end) site of pZErO-2, and the respective plasmids were designated pZTR06, pZTR07, and pZTR08. Then, pZTR06, pZTR07, and pZTR08 were digested with *Nhe*I, *Not*I, and *Spe*I, respectively, to isolate the ORFs of the *TR06*, *TR07*, and *TR08* genes, respectively. The gene fragments were sequentially inserted into the *Nhe*I (*TR06*), *Spe*I (*TR08*), and *Not* I (*TR07*) sites of pA3AXPC. The sequence of the resulting plasmid was confirmed and the plasmid was designated pA678.

The ORF fragment of the *TR09* gene was amplified by using the primers TR09-NheI-F and TR09-NheI-R (Supplementary Table S1). The ORF fragment of the *OMT* gene was amplified by using the primers OMT-NotI-F and OMT-NotI-R (Supplementary Table S1). The amplified fragments were cloned once into the *Eco*RV (blunt end) site of pZErO-2, and the plasmids were designated pZTR09 and pZOMT, respectively. Then, pZTR09 and pZOMT were digested with *Nhe*I and *Not*I, respectively, to isolate the ORFs of the TRAF09 gene and *OMT*, respectively. The gene fragments were sequentially inserted into the *Nhe*I (*TR09*) and *Not*I sites of pA3AXPC. The sequence of the resulting plasmid was confirmed and the plasmid was designated pA09OMT.

### Confirmation of Copy Numbers of Cluster Genes Introduced Into *A. oryzae*

Quantitative polymerase chain reaction (qPCR) was used to confirm the copy numbers of the cluster genes introduced into *A. oryzae*. The primer sets used to confirm the copy numbers are listed in Supplementary Table S1. Genomic DNA isolated from *A. oryzae* strains with all the cluster genes was used as the template. For reaction mixture preparation, KOD SYBR qPCR mix (Toyobo, Osaka, Japan) was used. Forty nanograms of genomic DNA extracted from each strain was applied to each reaction mixture (20 μL). The qPCR reaction was performed as described in the manufacturer’s instructions. The histone H4 gene was used as a normalization reference for extracted genomic DNA. The copy numbers of the introduced genes were calculated according to the number of PCR cycles, using *kexB* (Mizutani et al., 2004) as the standard for single-copy genes.

### Analysis of Transcription Levels of KK-1 Cluster Genes by Quantitative RT-PCR

qRT-PCR was performed as described previously (Yoshimi et al., 2013, 2015), with a slight modification regarding the qPCR mix. For reaction mixture preparation, KOD SYBR qPCR mix (Toyobo) was used, and the qPCR reaction was performed as described in the manufacturer’s instructions. Primer sets for quantifying the expression of KK-1 cluster genes are listed in Supplementary Table S1. The histone H4 gene was used as a normalization reference (internal control) for target gene expression ratios. Mycelia of each strain were obtained from three independent cultures and were used for RNA isolation and subsequent cDNA synthesis (three biological replicates). qRT-PCR analyses were then performed twice for each cDNA sample (two technical replicates).

### Marker Recycling in *A. oryzae*

The procedures used to create the self-excising Cre/*loxP*-mediated marker recycling system with mutated *lox* sequences have been described previously (Zhang et al., 2017). The parental strain of *A. oryzae* with adenine auxotrophy was transformed with the resulting expression plasmids in the presence of glucose, and then the transformants were cultured on medium containing xylose (1%) as the sole carbon source. Excision of both the selectable marker and the Cre expression construct was confirmed by PCR analysis of genomic DNA from the resultant colonies and their adenine auxotrophy.

### Production of KK-1 in *A. oryzae*

Because the genes for KK-1 biosynthesis that were introduced into *A. oryzae* were regulated by the *amyB* promoter, KK-1 production was assessed in YPM medium containing maltose (2%) as a carbon source for the promoter. Three replicates each of the control and the heterologous expression strains, which contained all cluster genes for KK-1, were cultured in 200 mL YPM in 500-mL Erlenmeyer flasks at 30°C and 160 rpm. After 4 days, the mycelia were removed by filtration through Miracloth (Merck KGaA, Darmstadt, Germany) and the culture supernatants were extracted twice with 200 mL of ethyl acetate. The extracts were combined and dehydrated with anhydrous sodium sulfate, and they were then evaporated and resuspended in 2 mL of acetonitrile. A 30-μL aliquot of each extract was separated in a Nexera-I LC-2040C HPLC system (Shimadzu, Kyoto, Japan) equipped with a column (CAPCELL PAK SG 120 5 μm, 4.6 mm × 250 mm, Shiseido, Tokyo, Japan). The KK-1 was eluted with a water–acetonitrile gradient containing 0.1% formic acid (50:50 for 5 min, to 2:98 for 15 min) at a flow rate of 1.0 mL/min, and was detected at a wavelength of 195 nm. Production of KK-1 was quantified by calculating the peak area of each extracted ion chromatogram (EIC) at a retention time of 17.9 min. KK-1 from *C. clavata* was used for external calibration. Liquid chromatography–mass spectrometry (LC-MS) analysis was performed with a Prominence UFLC (ultrafast liquid chromatograph) (Shimadzu) in combination with a 3200 Q TRAP MS (SCIEX, Tokyo, Japan). The HPLC conditions were the same as above, but the UV absorption peak of KK-1 was detected at a retention time of 18.7 min. The MS conditions were as follows: ionization mode, ESI (electrospray ionization) positive; turbo gas temperature, 650°C; ion spray voltage, 4 kV; curtain gas, 10 psi; ion source gas one, 70 psi; ion source gas two, 60 psi; scan range, *m/z* 500 to 1200.

The antifungal activity of the KK-1 produced by *A. oryzae* was assessed by using a test fungus, *B. cinerea* strain 26-1. Extracts derived from the culture supernatants were applied to 6-mm filter papers (Advantec, Tokyo, Japan), two of which were then placed on each petri dish. A small piece of potato dextrose agar on which the test fungus had been grown was placed on the center of each petri dish, which was then kept at 26°C for 3 days. Antifungal activity was evaluated as the average value of the inhibition halos of the test fungus.

## Results

### Reconstruction of the KK-1 NRPS Gene in *A. oryzae*

**FIGURE 2:**
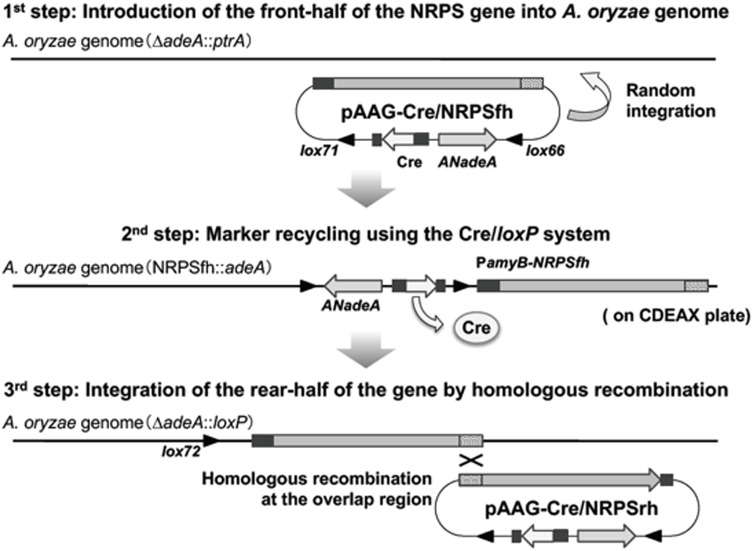
Strategy for reconstruction of the NRPS gene in *Aspergillus oryzae.* The first step (top) is the introduction of the front half of the NRPS gene into the *A. oryzae* genome. The second step (middle) is the strategy used for marker recycling using the Cre/*loxP* system. The third step (bottom) is integration of the rear half of the NRPS gene into the *A. oryzae* NRPSfh strain, resulting in reconstruction of the NRPS gene in *A. oryzae*.

The antifungal compound KK-1 (CAS No. 143380-71-6) is a non-ribosomal peptide originally identified from *C. clavata* (**Figure 1** and Yamaguchi et al., Unpublished). Non-ribosomal peptide synthesis is catalyzed by NRPS. The NRPS gene responsible for KK-1 biosynthesis in *C. clavata* is approximately 39 kb in size (Yamaguchi et al., Unpublished) and contains one very large coding sequence. Combination of the large coding sequence and the sequence of the vector part containing the Cre/*loxP* marker recycling unit would have resulted in a very large vector (exceeding 50 kb) for protoplast-based transformation of *A. oryzae*. Cloning vectors may not stably retain large inserts (>15 kb) (Anyaogu and Mortensen, 2015), and it is more difficult to purify large vectors than smaller ones. Therefore, we developed a strategy for reconstructing the NRPS gene in the *A. oryzae* genome (**Figure 2**). The gene was split into two fragments, NRPSfh and NRPSrh, both of which were approximately 20 kb in size and contained an overlap region of about 1 kb to enable concatenation of the fragments by homologous recombination in the *A. oryzae* genome (**Figure 2**). Construction of the two vectors containing the fragments NRPSfh and NRPSrh is described in the Section “Materials and Methods” (Supplementary Figures S1, S2). The resulting vectors were named pAAGC-NRPSfh and pAAGC-NRPSrh, respectively. First, the *A. oryzae* Δ*adeA* strain was transformed with the vector pAAGC-NRPSfh to introduce the front half of the NRPS gene, and integration of the vector into the genome was confirmed by PCR (data not shown). Then, the strain to which pAAGC-NRPSfh had been introduced was incubated on CDEAX medium, which contained adenine to complement the adenine auxotrophy of the resulting strain and xylose as a carbon source to induce the expression of *cre*. The induction of Cre recombinase production resulted in excision of the marker recycling unit located between the mutant *lox* sequences. Next, the resulting marker-recycled strain was used as the host strain for transformation with the vector pAAGC-NRPSrh to introduce the rear half of the NRPS gene. Correct concatenation of the two NRPS gene fragments by homologous recombination in the *A. oryzae* genome was confirmed by PCR (Supplementary Figure S3), and the concatenated DNA region was sequenced (data not shown). Eventually, two mutant lines of strains to which NRPS had been introduced, designated NRPS402 and NRPS403, were obtained by the above-described strategy for reconstruction of the NRPS gene in the *A. oryzae* genome; both NRPS402 and NRPS403 were used in the following experiments.

**FIGURE 3:**
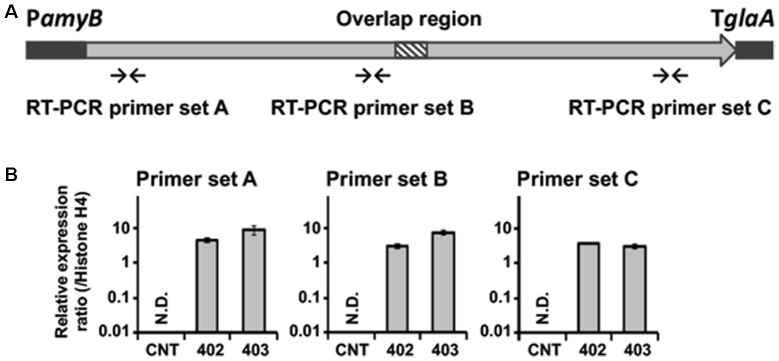
Expression of the NRPS gene in *A. oryzae*. **(A)** Arrows indicate regions of primer binding for qRT-PCR analyses. **(B)** Expression of the NRPS gene in the *A. oryzae* CNT strain and in strains NRPS-402 (402) and NRPS-403 (403) grown for 24 h in YPM medium. qRT-PCR was used to determine the levels of transcription of the NRPS gene by using primer sets A–C. Each value represents the ratio of expression to that of the histone H4 gene. Error bars represent standard deviations (three biological and two technical replicates). ND, not detected.

The results of our preliminary experiments revealed that the promoter of *C. clavata* did not work appropriately in *A. oryzae*. When the gene for the transcription factor of the *C. clavata* KK-1 cluster was overexpressed in *A. oryzae* after the introduction of the *OMT* gene of the cluster, an appropriate reaction—namely, *OMT* gene expression corresponding to the transcript levels of the transcription factor—was not observed (data not shown). Therefore, NRPS gene expression was designed to be driven instead by the *amyB* promoter in *A. oryzae*. To determine whether the reconstructed NRPS gene was expressed in *A. oryzae*, we analyzed the transcript level of NRPS in the NRPS402 and NRPS403 strains by qRT-PCR (**Figure 3**). The strains were cultured in YPM medium, which contained maltose as an inductive substrate for the *amyB* promoter, and mRNA was extracted from the mycelia of *A. oryzae* CNT and the strain into which NRPS had been introduced. cDNA was then synthesized from the mRNA and used as a template for quantitative RT-PCR. Whereas the transcript of the NRPS gene was undetectable in the CNT strain using any of three primer sets located in the coding region (near the 5′- or 3′-end or in the middle; **Figure 3A**), the transcript was readily detectable in both NRPS-transformed strains grown in YPM medium for 24 h (**Figure 3B**).

### Simultaneous Introduction of Seven Cluster Genes Into the Genome

**FIGURE 4:**
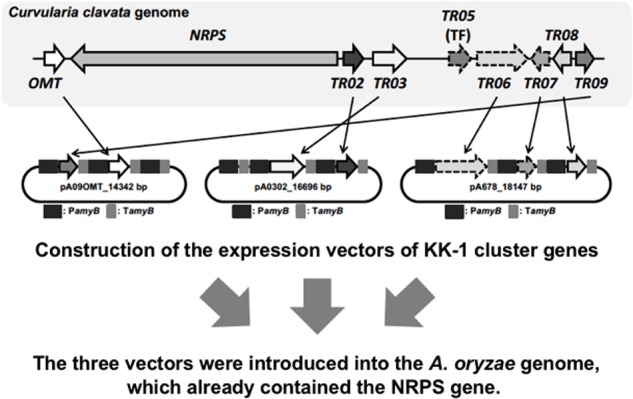
Strategy for introducing all genes involved in KK-1 biosynthesis. Shown is the cloning strategy used to introduce the seven cluster genes into the plasmid pA3AXPC, along with the resulting vectors, pA09OMT, pA0302, and pA678.

Because we had developed a strategy by which all cluster genes were driven by the *amyB* promoter in *A. oryzae*, it seemed that introducing a transcription factor into the cluster was not necessary. Therefore, among the nine cluster genes involved in KK-1 biosynthesis, seven (apart from NRPS and the transcription-factor-encoding gene), which were named *OMT*, *TR02*, *TR03*, *TR06*, *TR07*, *TR08*, and *TR09* (see **Figure 4**), were introduced into the *A. oryzae* strain with NRPS. To introduce these remaining seven genes, we first induced excision of the marker recycling unit in one of the strains to which NRPS had been introduced (NRPS402) by using the same methods as described above. Then, three different vectors, each containing two or three genes (for construction details see the section “Materials and Methods”) were transformed into the marker-recycled strain. We initially attempted sequential introduction of the vectors, but eventually the three vectors were simultaneously introduced into the strain. Checking of more than 100 of the transformants confirmed integration of the remaining seven genes into the genome in six of the transformants (data not shown). These transformants were collectively named the *A. oryzae* KK-1 strains.

### Expression Profiles of KK-1 Cluster Genes in *A. oryzae*

**FIGURE 5:**
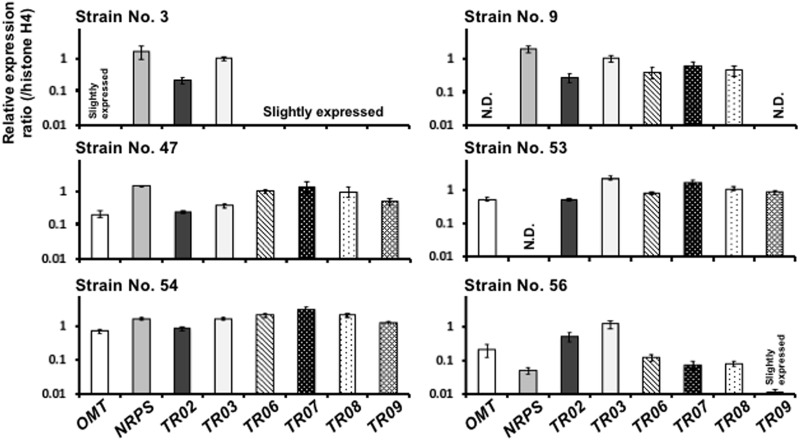
Expression of all genes introduced into *A. oryzae*. Expression of the genes required for KK-1 biosynthesis in *A. oryzae* strains 3, 9, 47, 53, 54, and 56 grown for 24 h in YPM medium. qRT-PCR was used to determine the levels of transcription of the indicated genes by using gene-specific primers. Each value represents the ratio of expression to that of the histone H4 gene in each strain. Error bars represent standard deviations (three biological and two technical replicates). ND, not detected.

To determine whether all the genes heterologously introduced into *A. oryzae* were expressed, we analyzed the transcript levels of the introduced genes in the *A. oryzae* KK-1 strains by qRT-PCR (**Figure 5**). Because all of the genes were designed to be driven by the *amyB* promoter in *A. oryzae*, the *A. oryzae* KK-1 strains were cultured in YPM medium, as in the analysis of NRPS gene expression, and mRNA was extracted from the mycelia of CNT and all of the strains into which the genes had been introduced. cDNA was then synthesized from the extracted mRNA and used as a template for qRT-PCR. In the *A. oryzae* CNT strain grown in YPM medium for 24 h, the transcripts of all genes were undetectable (data not shown). Although the transcripts of several genes were not detectable, or barely detectable, in *A. oryzae* KK-1 strains 3, 9, 53, and 56 (**Figure 5**), the transcripts of all genes were detected in the remaining two strains (numbers 47 and 54) grown in YPM medium for 24 h (**Figure 5**). High transcript levels of all genes were detected in strain 54 (**Figure 5**), and the transcript levels were comparable to that of histone H4 (**Figure 5**). The results of statistical analyses by the multiple comparison of transcript levels for each gene among the strains are shown in Supplementary Figure S4. The transcript levels of most genes were significantly different among strains. These results suggested that there were variations in transcript levels of the genes, depending on the strain.

To elucidate the factors involved in this instability of transcript levels, the copy numbers of the introduced genes were determined by qPCR. Most genes were multiply introduced into strain 54 (**Table 1**). Although single-copy introduction of all genes was detected in strain 3 (**Table 1**), several genes were only slightly expressed in strain 3 (**Figure 5**). On the other hand, the NRPS gene was not detected in strain 53 (**Table 1**), and this led to non-detection of the NRPS transcript (**Figure 5**), suggesting that deletion of the NRPS gene occurred during the process of culture for DNA extraction to confirm the copy number.

**Table 1:** Copy numbers of genes involved in KK-1 biosynthesis that were introduced into *Aspergillus oryzae*.

		Copy number							
	
Strain number	*O*-MT	NRPS	TRAF02	TRAF03	TRAF06	TRAF07	TRAF08	TRAF09
3	1	1	1	1	1	1	1	1
9	ND	1	1	1	1	1	1	ND
47	1	1	1	1	1	1	1	1
53	3	ND	1	1	1	>3	3	3
54	>3	1	>3	2	>3	>3	>3	>3
56	2	†	2	1	†	†	†	†


**ND, not detected. ^†^Calculated to be less than one copy, suggesting heterokaryosis of strain 56 and poor population of the nucleus into which the genes were introduced.**

### Analysis of KK-1 Production in *A. oryzae*

Preliminarily, we analyzed the qualitative activity against *B. cinerea* of extracts derived from the *A. oryzae* KK-1 strains (Supplementary Figure S5). After 4 days of culture in YPM medium, the culture supernatants were extracted with acetonitrile, and the extracts were used in a halo assay on petri dishes. The extracts from strains 3, 9, 54, and 56 showed strong activity against *B. cinerea* (Supplementary Figure S5).

**FIGURE 6:**
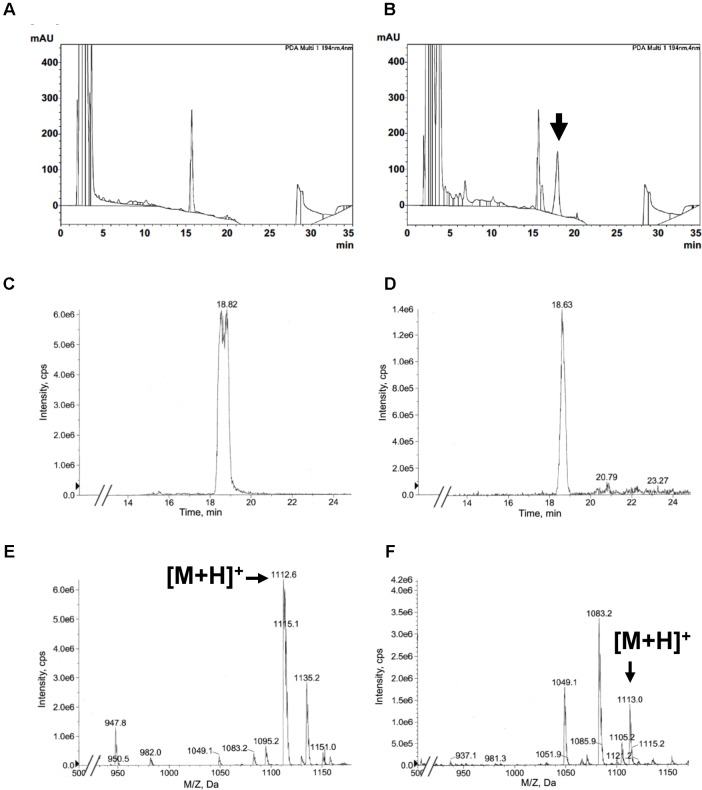
KK-1 production, and confirmation of production, in *A. oryzae*. **(A,B)** HPLC UV spectra of **(A)** extract of supernatant from culture of the *A. oryzae* CNT strain and **(B)** extract of supernatant from culture of strain 54, into which all genes were successfully introduced. UV spectrum of the peak indicated by the arrow is identical to that of standard KK-1 derived from *Curvularia clavata*. **(C,D)** Extracted ion chromatograms (*m/z* 1113). **(C)** Standard KK-1 derived from *Curvularia clavata*. **(D)** Extract of supernatant from culture of *A. oryzae* strain 54. **(E,F)** Mass spectra of KK-1. **(E)** Standard KK-1 derived from *Curvularia clavata*. **(F)** Extract of supernatant from culture of *A. oryzae* strain 54.

Next, we assessed the productivity of KK-1 derived from *A. oryzae* KK-1 strain 54, which showed high transcript levels of all genes, and we estimated KK-1 production by using the halo assay. After 4 days of culture in YPM medium, the culture supernatants were extracted with ethyl acetate and the extract was analyzed by HPLC and LC-MS (**Figure 6**). Whereas the peak corresponding to KK-1 was not detected in the extract derived from the *A. oryzae* CNT strain or from the medium only (**Figure 6A** and data not shown), a peak corresponding to KK-1 was clearly detected in the extract derived from *A. oryzae* KK-1 strain 54 (**Figure 6B**). LC-MS analysis was performed on strain 54 and on strain 53, in which the transcript of NRPS was not detected. Extraction ion chromatograms (EICs) of *m/z* 1113 corresponding to the mass of KK-1 in positive mode are shown in **Figures 6C,D**, and mass spectra are shown in **Figures 6E,F**. An EIC peak derived from KK-1 (**Figure 6C**: standard KK-1) was clearly detected in *A. oryzae* KK-1 strain 54 (**Figure 6D**) but not in strain 53 (data not shown); the detected peak had a fragment ion mass spectrum similar to that of the KK-1 standard (**Figures 6E,F**). These results revealed that KK-1 was produced by *A. oryzae* KK-1 strain 54 concurrently with the expression of the cluster genes. The mean amount of KK-1 produced was 4 mg/L, as calculated from the peak area. In KK-1 strains 3, 9, and 56, extracts of which showed antifungal activity against *B. cinerea*, an EIC peak derived from KK-1 and a fragment ion mass spectrum similar to that of KK-1 were detected (Supplementary Figure S6), although their signals in the extract derived from strain 9 were comparably weak (Supplementary Figures S6C,D).

## Discussion

Here, we succeeded in reconstructing a large NRPS gene in the *A. oryzae* genome (**Figure 2** and Supplementary Figure S3) and confirmed the gene’s expression (**Figure 3**), which was designed to be driven by the *amyB* promoter in *A. oryzae*. This result suggested that expression of the NRPS gene was successfully driven by the *amyB* promoter. Incidentally, according to the genome sequence database of *A. oryzae* (Machida et al., 2005; AspGD^1^) and our own knowledge, the largest gene in *A. oryzae* is AOR_1_1634054, which encodes an NRPS (6885 amino acid residues) and includes an ORF of 20,892 bp, interrupted by four introns. Therefore, our results indicated that a gene twice as large as the largest gene in *A. oryzae* could be successfully transcribed by the *amyB* promoter.

To introduce the remaining seven cluster genes (i.e., excluding the genes encoding NRPS and the transcription factor) into the *A. oryzae* genome, we constructed three different vectors, each containing two or three genes. Initially, we introduced them sequentially and repeated the marker recycling, and integration of the vector into the genome was confirmed each time by PCR (data not shown). Unexpected results were obtained: deletion of some of the introduced genes was observed. A possible explanation for these results is that loop-out of the genes occurred between the homologous sequences for the promoter or terminator (i.e., the *amyB* promoter or terminator). Therefore, we redeveloped our strategy for introducing the remaining seven genes: the three vectors with the remaining seven genes were simultaneously introduced into the genome of the parental strain (**Figure 4**). In general, co-transformation of non-selected plasmids leads to increased efficiencies of transformation in filamentous fungi (Ruiz-Díaz, 2002). In addition, Tagami et al. (2013, 2014) reported that co-transformation led to the simultaneous introduction of two vectors in one round of transformation using selection for only one marker. Therefore, we considered that there would be no problem in using the same selectable marker (i.e., the *adeA* marker)—for example, in cases where the number of selectable markers is limited. Simultaneous transformation of multiple vectors was thus efficient in the heterologous introduction of multiple genes into *A. oryzae*.

Next, we analyzed the transcript levels of the genes in *A. oryzae* and found that there were variations in transcript levels, depending on the strain, despite the successful introduction of all the genes (**Figure 5** and Supplementary Figure S4). The variations in the transcript levels of the genes seemed to depend on the location of vector integration into the genome. These results suggested that control of the locus of vector integration and of the copy numbers of the vectors was difficult in simultaneous transformation, and that the vectors were randomly integrated into the genome in the resulting transformants. On the other hand, the NRPS gene was not detected in strain 53 (**Table 1**), and this led to non-detection of the NRPS transcript (**Figure 5**), suggesting that deletion of the NRPS gene occurred during the process of culture for DNA extraction to confirm the copy number. Because NRPS genes generally have repeated modules with adenylation, condensation, and thiolation domains, and this causes DNA sequence similarity among modules in the gene, the loop-out and resulting excision caused by the repeating sequences might have been a problem in NRPS introduction.

By using halo assay on petri dishes, we confirmed antifungal activity against *B. cinerea* in the extracts from strains 3, 9, 54, and 56. In addition, KK-1 production was confirmed by LC-MS analysis of the strains (**Figure 6** and Supplementary Figure S6), although only weak signals corresponding to KK-1 were detected in the extract derived from strain 9. These results were partly inconsistent with the results of our transcript analysis of strains 3, 9, and 56 (**Figure 5**). A possible explanation for this is that only small amounts of the transcripts of the genes were enough for KK-1 production in strains 3, 9, and 56, or that a compound produced without, or with extremely low levels of, *OMT* and *TR09* in strain 9 had antifungal activity as well (see Supplementary Figure S5).

The clearest heterologous production of KK-1 was detected in the culture broth of *A. oryzae* strain 54, which showed high transcript levels of all genes, suggesting that expression of the cluster genes was necessary and sufficient for the heterologous production of KK-1 in *A. oryzae*. On the other hand, the amount of KK-1 produced by strain 54 was markedly lower than that from the original producer, the *C. clavata* wild-type strain (50 mg/L), suggesting that there are unknown factors limiting productivity in *A. oryzae*. In addition, the antifungal activity of the extract derived from strain 47 was barely detectable by halo assay (Supplementary Figure S5), regardless of our finding that all gene transcripts were observed in this strain (**Figure 5**). It is thus reasonable to hypothesize that KK-1 productivity was limited by factors other than gene expression—for example, an insufficient supply of precursor amino acids as substrates for KK-1 biosynthesis in *A. oryzae*.

Among the various fungal species, *Aspergillus* species are the most commonly used hosts for heterologous production of secondary metabolites. Munawar et al. (2013) used *A. oryzae* to heterologously express the *FNS1* gene, which is approximately 14 kb long and is responsible for synthesis of the siderophore ferrirhodin in *Fusarium sacchari*. Sakai et al. (2012) reported that heterologous introduction of a large gene cluster from *Monascus pilosus* [the gene cluster was 42 kb long and was involved in monacolin K (MK) biosynthesis] resulted in the successful production of MK in *A. oryzae*. They introduced the cosmid 12–33, which contains the majority of the MK gene cluster, and the plasmid sCnDmokB, which contains *mokB* (which is additionally required for MK biosynthesis in combination with the rest of the MK biosynthetic genes in 12–33), to *A. oryzae*. They also attempted the overexpression of *laeA*, which encodes a global regulator for the production of secondary metabolites (Bok and Keller, 2004; Amare and Keller, 2014), resulting in the heterologous production of MK in *A. oryzae. A. nidulans* is a model filamentous fungus and has also been used as a heterologous host for secondary metabolites from other fungal species. Nielsen et al. (2013) demonstrated the stepwise transfer of two vectors into *A. nidulans*. The two vectors contained the entire gene cluster responsible for geodin biosynthesis in *A. terreus*; it is 25 kb long and contains a total of 13 ORFs. Recently, Bok et al. (2015) reported that 15 fungal artificial chromosomes, which ranged from 70 to 150 kb in length and contained gene clusters for secondary metabolites in *A. terreus*, were successfully transformed into *A. nidulans*. Here, we also succeeded in the heterologous introduction of a large gene cluster, which was approximately 75 kb long. In some cases of heterologous expression of cluster genes, the promoter controlling the expression of the gene for cluster-specific transcription factor has been swapped for a strong constitutive promoter, resulting in successful heterologous production (Nielsen et al., 2013; Yin et al., 2013). However, as described above, the promoter of *C. clavata* did not work appropriately in *A. oryzae*, regardless of overexpression of the transcription factor in the KK-1 gene cluster. Therefore, we adopted a method by which all genes in the large cluster responsible for KK-1 biosynthesis were controlled by the inducible *amyB* promoter and were successfully introduced into *A. oryzae*, resulting in the successful production of KK-1. To our knowledge, such an elaborate attempt has not been reported before in the heterologous production of secondary metabolites in fungi. Our heterologous expression system, including the *in vivo* reconstruction of a large gene in the *A. oryzae* genome and the use of vectors for multiple gene cloning with the Cre/*loxP* marker-recycling system, could provide an efficient approach to linking in large gene clusters to create novel bioactive compounds.

## Author Contributions

AY, KK, TF, MM, and KA conceived and designed the experiments. AY carried out the construction of the fungal strains. AY and SY performed the essential experiments and analyzed the data. AY, SY, KK, KG, and KA wrote the paper. All authors discussed the results and commented on the manuscript.

## Conflict of Interest Statement

The authors declare that the research was conducted in the absence of any commercial or financial relationships that could be construed as a potential conflict of interest.

## Footnotes

1 http://www.aspergillusgenome.org

## Supplementary Material

**FIGURE S1 |** Schematic illustration of construction of the vector for the front half of the NRPS gene. The front half of the NRPS gene from *Curvularia clavata* (approximately 20 kb) was amplified by using PrimeSTAR GXL DNA polymerase and the primers NRPS_FH-F-NotI and NRPS_FH-R-NotI (see **Supplementary Table S1**). Each primer was designed to introduce a *Not*I site. *Curvularia clavata* genomic DNA was used as the template. The amplified fragment was cloned once into the *Eco*RV (blunt end) site of pZErO-2. Then, the fragment containing the front half of the NRPS gene was digested with *Not*I, corrected, and inserted into the *Not*I site of pAAG-Cre, which contained the *amyB* promoter, *glaA* terminator, and *Cre*/mutant *lox* marker recycling system (Zhang et al., 2017). The sequences of the resulting plasmid (pAAGC-NRPSfh) were confirmed, and the plasmid was used to transform the *A. oryzae* Δ*adeA* strain (*sC*^-^, *niaD*^-^, Δ*ligD*::*sC*, Δ*adeA*::*ptrA*).

**FIGURE S2 |** Schematic illustration of construction of the vector for the rear half of the NRPS gene. Three fragments, designated NRPS rh fragment A, NRPS rh fragment B, and NRPS rh fragment C, were amplified by using PrimeSTAR GXL DNA polymerase with the primers NRPS_RH-IF1-Not I-F and NRPS_RH-IF1-R (for fragment A), NRPS_RH-IF2-F and NRPS_RH-IF2-R (for fragment B), and NRPS_RH-IF3-F and NRPS_RH-IF3-Not I-R (for fragment C). The amplified fragments were purified by gel extraction and fused to the *Not*I-digested pAAG-Cre by using an In-Fusion HD Cloning kit. The sequences of the resulting plasmid (pAAGC-NRPSrh) were confirmed, and it was used to transform the NRPSfh-integrated strain of *A. oryzae*.

**FIGURE S3 |** Confirmation of reconstruction of the NRPS gene in *Aspergillus oryzae*. **(A)** Arrows indicate the regions amplified by PCR for confirmation of the presence of the integrated NRPS gene. Fragment f contained the concatenated region of the reconstruction of the NRPS gene. **(B)** Images of electrophoresis of PCR products. Numbers 1 to 5 indicate the strains NRPS101, 102, 401, 402, and 403, respectively. P, positive control (*Curvularia* genome). N, negative control (*Aspergillus oryzae* CNT genome).

**FIGURE S4 |** Comparison of transcript levels of each gene among strains of *Aspergillus oryzae*. Levels of transcription of indicated genes were determined by means of qRT-PCR using gene-specific primers (**Supplementary Table S1**). Each value represents the ratio of expression to that of the histone H4 gene in each strain. Data are means of three biological and two technical replicates, with standard deviations. Different letters indicate statistically significant differences among strains (*P* < 0.05, Tukey’s multiple comparison test).

**FIGURE S5 |** Activity of extract derived from strains of *Aspergillus oryzae* against *Botrytis cinerea* test strain. The test fungus *B. cinerea* was inoculated into the center of the petri dishes. Extracts from the culture supernatants were then dropped onto paper disks, which were then placed on the petri dishes. The fungus was grown at 26°C for 3 days. Antifungal activity was evaluated as the average values of the inhibition halos of the test fungus. ACN, acetonitrile.

**FIGURE S6 |** Extracted ion chromatograms (*m/z* 1113) and mass spectra of KK-1 derived from strains 3, 9, and 56. **(A,C,E)** Extracted ion chromatograms (*m/z* 1113) of extracts of supernatants from culture of *Aspergillus oryzae* strains 3 **(A)**, 9 **(C)**, and 56 **(E)**. **(B,D,F)** Mass spectra of KK-1 in extracts of supernatants from culture of *A. oryzae* strains 3 **(B)**, 9 **(D)**, and 56 **(F)**.

**TABLE S1 |** Primers used in this study.
