# Supplementary figures and images for: Heterologous Production of a Novel Cyclic Peptide Compound, KK-1, in Aspergillus oryzae
Source: Front Microbiol. 2018 Apr 9;9:690. doi: 10.3389/fmicb.2018.00690 (PMC5900794; doi:10.3389/fmicb.2018.00690)

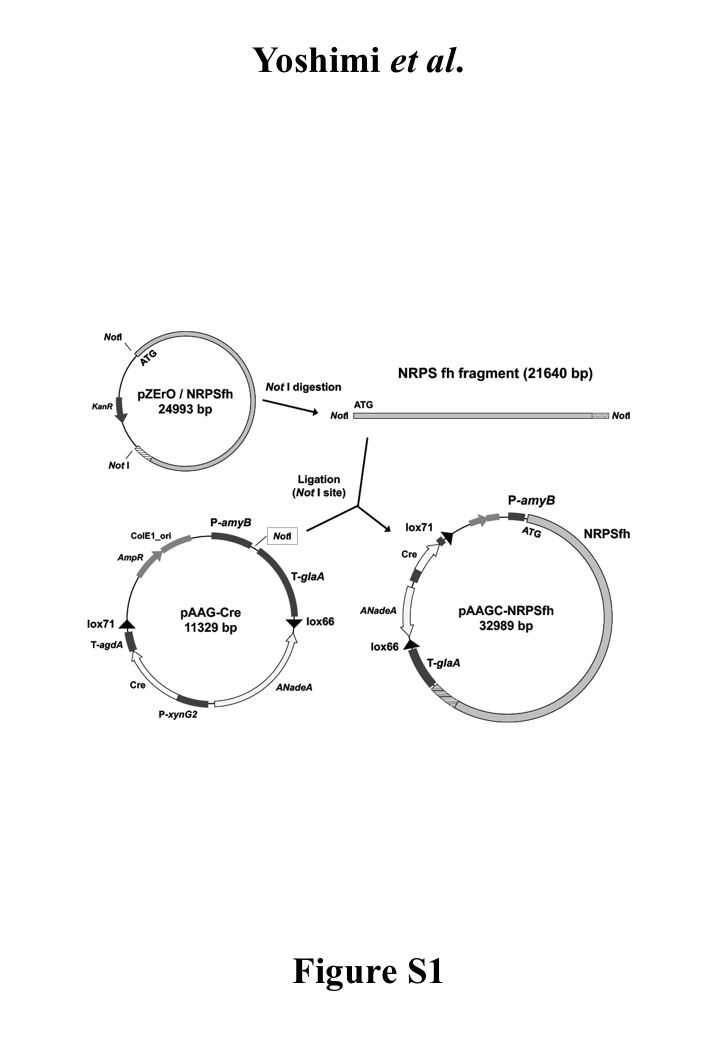

Supplement: Supplementary file 1 [file Image_1.TIFF]

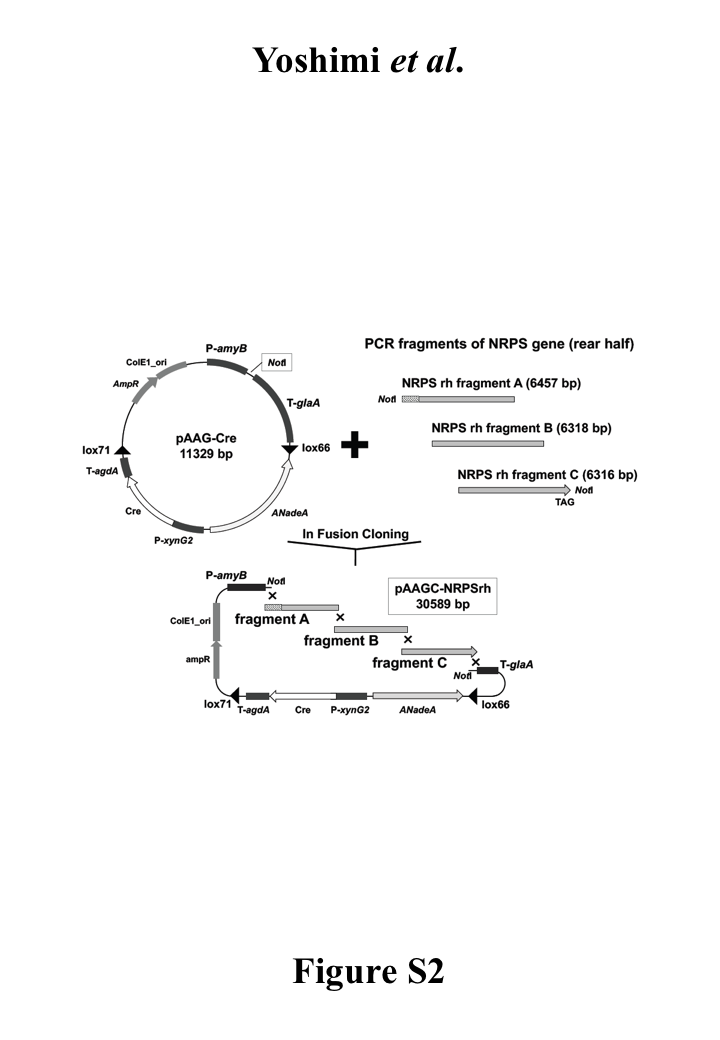

Supplement: Supplementary file 2 [file Image_2.TIFF]

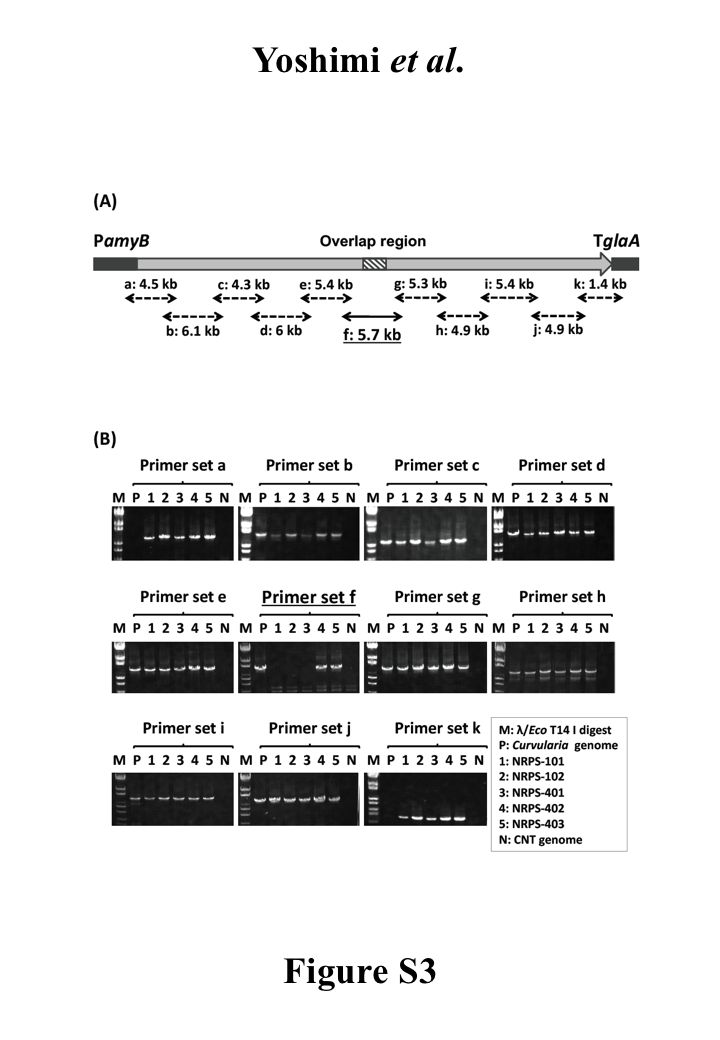

Supplement: Supplementary file 3 [file Image_3.TIFF]

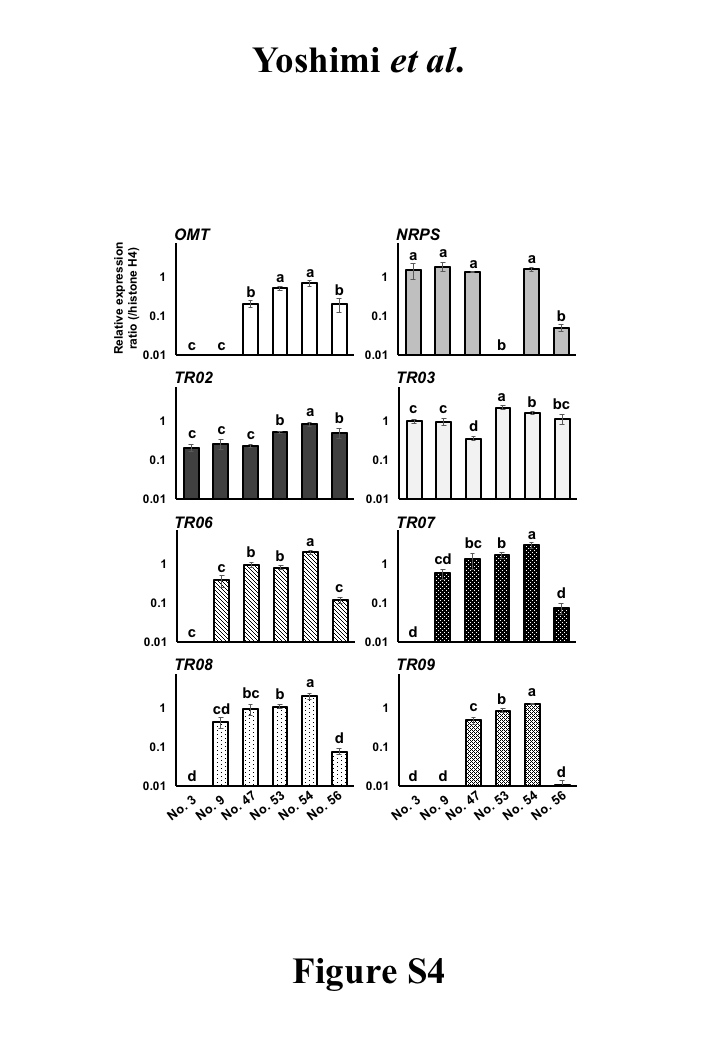

Supplement: Supplementary file 4 [file Image_4.TIFF]

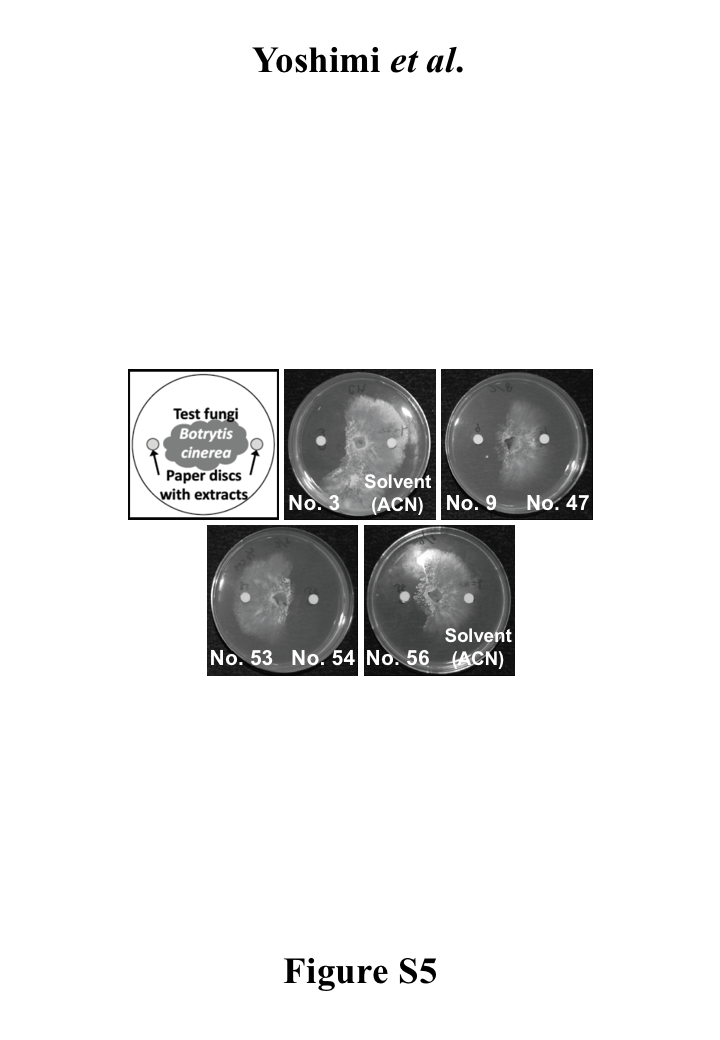

Supplement: Supplementary file 5 [file Image_5.TIFF]

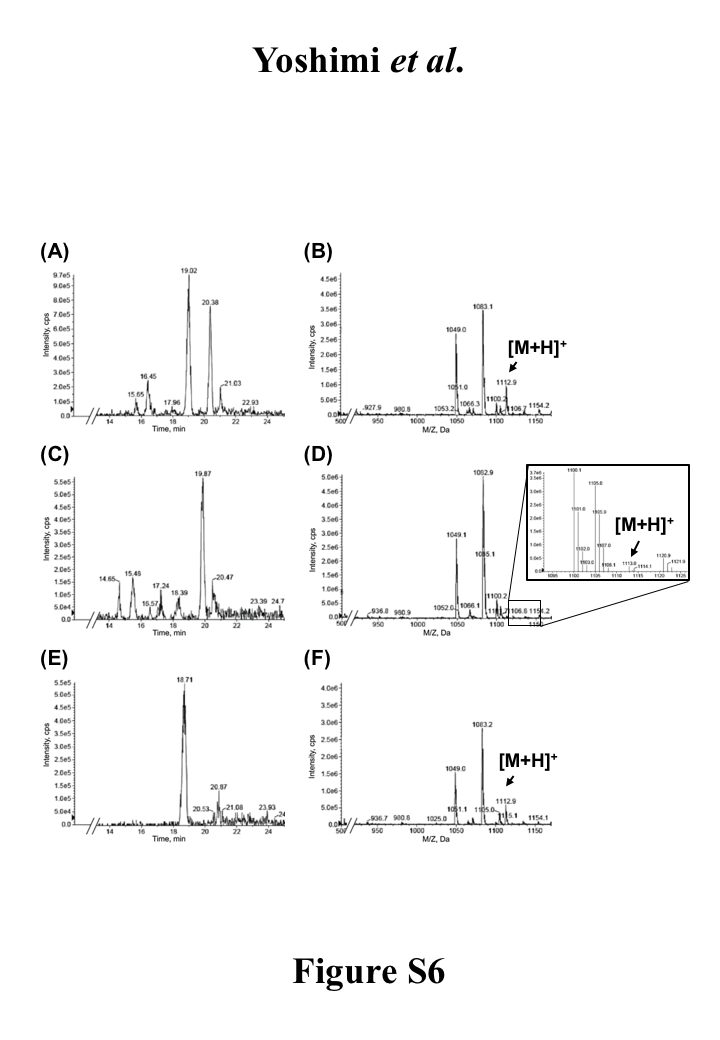

Supplement: Supplementary file 6 [file Image_6.TIFF]
