# Supplementary material for: Heterologous Production of a Novel Cyclic Peptide Compound, KK-1, in Aspergillus oryzae
Source: Front Microbiol. 2018 Apr 9;9:690. doi: 10.3389/fmicb.2018.00690 (PMC5900794; doi:10.3389/fmicb.2018.00690)
Supplement: Supplementary file 7 [file Table_1.DOCX]

Table S1 Primers used in this study

| Purpose | Name | Sequence (5’-3’) |  |
| --- | --- | --- | --- |
| Cloning of the gene for NRPS | | | |
|  | NRPS_FH-F-Not I | TCGACAAGCTTGCGGCCGCCACGTGACTAGTATGGCCAGCGACATCAATACTCATCCAG |  |
|  | NRPS_FH-R-Not I | ACTAGTCACGTGGCGGCCGCGGCGCGCCAAGATCGTCTTGCTGTACG |  |
|  | NRPS_RH-IF1-Not I-F | GATGCGCTAGCGGCCGCGAAGTGGTCCTTGTCGCTGGTGAC |  |
|  | NRPS_RH-IF1-R | TGCCGTTCGCATTCATAGGCATCTCGTC |  |
|  | NRPS_RH-IF2-F | TGAATGCGAACGGCAAGGTTGACAG |  |
|  | NRPS_RH-IF2-R | CTTGGTTGCTGGCTTCGTCGTTGTC |  |
|  | NRPS_RH-IF3-F | AAGCCAGCAACCAAGTCGAAGATTG |  |
|  | NRPS_RH-IF3-Not I-R | GTCACTAGTGCGGCCGCCTATTTTTGCAAGATCTTGTTCAAAC |  |
|  |  |  |  |
| Confirmation of reconstruction of the NRPS gene | | |  |
|  | PamyB-F | CGCTTACCGATTACGTTAGGGCTGA |  |
|  | NRPS_setA-R | TGGAGATGTGCAATAGGCGTCTGTG |  |
|  | NRPS_setB-F | TATTGCACATCTCCAGCTGACTGATG |  |
|  | NRPS_setB-R | GTTTAAACGCGGCCGCCTCAGTCTCATTACGGGCACCAACACGT |  |
|  | NRPS_setC-F | GTTTAAACGCGGCCGCGCAATCGATGCCTCTGCTGTTGGTAACG |  |
|  | NRPS_setC-R | GTTTAAACGCGGCCGCACCTCAGCCAATTCAATGCGATGTCCTC |  |
|  | NRPS_setD-F | GTTTAAACGCGGCCGCCTGCTTGAAGTTGAGCAGCCTCAATATC |  |
|  | NRPS_setD-R | GTTTAAACGCGGCCGCGTTGAATACGCTCCGCAGCTGTTTCGA |  |
|  | NRPS_setE-F | GTTTAAACGCGGCCGCAATGAAGCCACCAATCAAGTAGCTGGATG |  |
|  | NRPS_FH-R-Not I | ACTAGTCACGTGGCGGCCGCGGCGCGCCAAGATCGTCTTGCTGTACG |  |
|  | NRPS_setF-F | CAGACGGCTGGTCAGTCGATGTTCT |  |
|  | NRPS_setF-R | CGATACGAGCAGATGGTACGTTGAC |  |
|  | NRPS_setG-F | GAGGAAGAATTGCTCGTCGACCCAACCTTC |  |
|  | NRPS_setG-R | GTTTAAACGCGGCCGCCTCACAGAGGAGAGCCTCTATCTCGTTG |  |
|  | NRPS_setH-F | CATCTGCTCGTATCGAGGCGATCCT |  |
|  | NRPS_setH-R | GTCACTAGTGCGGCCGCGAGGGCTTTGCTATCCAGAACGGTG |  |
|  | NRPS_setI-F | GATGCGCTAGCGGCCGCCAGCTTGATTACTGGACGAAGAACC |  |
|  | NRPS_setI-R | CTTGGTTGCTGGCTTCGTCGTTGTC |  |
|  | NRPS_setJ-F | AAGCCAGCAACCAAGTCGAAGATTG |  |
|  | NRPS_setJ-R | ATGCTGTTCGTTGTCGACAAGGTTC |  |
|  | NRPS_setK-F | GACAACGAACAGCATCCCCTTCCAG |  |
|  | TglaA-R | GCCTGCAGGCCCGGGTCACTAGTGCG |  |
|  |  |  |  |
| Cloning of the seven cluster genes | | | |
|  | OMT-Not I-F | ATTTGCGGCCGCATGGACCCGAGACAGTCACGGATC |  |
|  | OMT-Not I-R | ATTTGCGGCCGCTTATGGTGTGGTGGGTTGCCATTC |  |
|  | TR02-Spe I-F | GGACTAGTATGACTGAACCCACATGGAA |  |
|  | TR02-Spe I-R | GGACTAGTTTAATAATCTACTTCAAGCAC |  |
|  | TR03-Not I-F | ATAAGAATGCGGCCGCATGGCGTTGCAAGAGCG |  |
|  | TR03-Not I-R | ATAAGAATGCGGCCGCTCAAGATGGGAAAGCCGCTG |  |
|  | TR06-Nhe I-F | CTAGCTAGCATGAGTGCTATCGAGCTGC |  |
|  | TR06-Nhe I-R | CTAGCTAGCTCAGCGATTGAGGGCCTGG |  |
|  | TR07-Not I-F | ATAAGAATGCGGCCGCATGAAGCTCACCGTTTTCAG |  |
|  | TR07-Not I-R | ATAAGAATGCGGCCGCTCAGAGCCGCGCCAAC |  |
|  | TR08-Spe I-F | GGACTAGTATGACGAAAAGGGAAAGCAAC |  |
|  | TR08-Spe I-R | GGACTAGTCTACGCGTTTTCTTTCGAC |  |
|  | TR09-Nhe I-F | CTAGCTAGCATGGAGAGCGAAGACAATCC |  |
|  | TR09-Nhe I-R | CTAGCTAGCTCAGCAGTATCCCATCGG |  |
|  |  |  |  |
| Confirmation of copy numbers of the introduced genes | | |  |
|  | kexB-RT-F | GCGACATCAGTGTGGAGTTG |  |
|  | kexB-RT-R | GACCGTCCACTTTCCAACAC |  |
|  | NRPS-RT2-F | GACCGTTACAGCGAGTTCAG |  |
|  | NRPS-RT2-R | CTGAATTCCTCGCACAGAAC |  |
|  | OMT-RT-F | ACGTTCAAGACCTTCCAG |  |
|  | OMT-RT-R | GTTCCGGATGATTTGCAG |  |
|  | TR02-RT-F | GCCCTACTAGATCTGACCAC |  |
|  | TR02-RT-R | GCTGTTACCTTTTCCTCCTC |  |
|  | TR03-RT-F | CATCGCTGCAGACTTAGATG |  |
|  | TR03-RT-R | GCATTCGTCCCACAGCATAC |  |
|  | TR06-RT-F | ACGTCCAGGAAGCTATCGAG |  |
|  | TR06-RT-R | ATTGAGGGCCTGGGCTTGAC |  |
|  | TR07-RT-F | GTGATGAAGGCGCTGAAGAG |  |
|  | TR07-RT-R | CTCCGCAATTTCCGTGAGTG |  |
|  | TR08-RT-F | TGACTCTATGGTGGATGGTG |  |
|  | TR08-RT-R | CCTTGTTCAAGTGCCAGTAG |  |
|  | TR09-RT-F | GATTCCGTCACGAGACACTG |  |
|  | TR09-RT-R | AGTATCCCATCGGGCAACAG |  |
|  |  |  |  |
| Quantitative RT-PCR | | |  |
|  | Histone H4-RT-F | CAAGCGTATCTCTGCCATGA |  |
|  | Histone H4-RT-R | CACCGAAACCGTAGAGGGTA |  |
|  | NRPS-RT1-F | GACGCCACGAACGCATAGAC |  |
|  | NRPS-RT1-R | TTCCCAGAGAGGTAGATCGAC |  |
|  | NRPS-RT2-F | GACCGTTACAGCGAGTTCAG |  |
|  | NRPS-RT2-R | CTGAATTCCTCGCACAGAAC |  |
|  | NRPS-RT3-F | GAAGTTGAGAACGCCATGCT |  |
|  | NRPS-RT3-R | GATGCGAGATGGGAGCATGT |  |
|  | OMT-RT-F | ACGTTCAAGACCTTCCAG |  |
|  | OMT-RT-R | GTTCCGGATGATTTGCAG |  |
|  | TR02-RT-F | GCCCTACTAGATCTGACCAC |  |
|  | TR02-RT-R | GCTGTTACCTTTTCCTCCTC |  |
|  | TR03-RT-F | CATCGCTGCAGACTTAGATG |  |
|  | TR03-RT-R | GCATTCGTCCCACAGCATAC |  |
|  | TR06-RT-F | ACGTCCAGGAAGCTATCGAG |  |
|  | TR06-RT-R | ATTGAGGGCCTGGGCTTGAC |  |
|  | TR07-RT-F | GTGATGAAGGCGCTGAAGAG |  |
|  | TR07-RT-R | CTCCGCAATTTCCGTGAGTG |  |
|  | TR08-RT-F | TGACTCTATGGTGGATGGTG |  |
|  | TR08-RT-R | CCTTGTTCAAGTGCCAGTAG |  |
|  | TR09-RT-F | GATTCCGTCACGAGACACTG |  |
|  | TR09-RT-R | AGTATCCCATCGGGCAACAG |  |
|  |  |  |  |
